# Effect of treatment with an overheated dry-saturated steam vapour disinfection system on multidrug and extensively drug-resistant nosocomial pathogens and comparison with sodium hypochlorite activity

**DOI:** 10.1186/s13104-015-1534-9

**Published:** 2015-10-09

**Authors:** Maria Bagattini, Raffaella Buonocore, Maria Giannouli, Dario Mattiacci, Rossella Bellopede, Nicola Grimaldi, Antonio Nardone, Raffaele Zarrilli, Maria Triassi

**Affiliations:** Dipartimento di Sanità Pubblica, Università degli Studi di Napoli “Federico II”, Via S. Pansini n.5, 80131 Naples, Italy; Anestesia, Rianimazione e Terapia Intensiva, Seconda Università degli Studi di Napoli, Naples, Italy

**Keywords:** Steam vapour disinfection system, Chlorine or hypochlorite disinfection, Nosocomial pathogens, Multidrug and extensively drug-resistant bacteria

## Abstract

**Background:**

The development of portable steam generators has made disinfection of the environment more practical. This study assessed the “in vitro” ability of an overheated dry-saturated steam vapour system to kill multidrug and extensively-drug resistant nosocomial pathogens, defining the antimicrobial spectrum and the contact times compared with the activity of sodium hypochlorite.

**Methods:**

The antibacterial efficacy of the overheated dry-saturated steam vapour system and of sodium hypochlorite against nosocomial pathogen isolates: extensively drug-resistant *Acinetobacter baumannii*, *Pseudomonas aeruginosa*, carbapenemase-producing *Klebsiella pneumoniae*, methicillin-resistant *Staphylococcus aureus*, high-level aminoglycoside-resistant *Enterococcus faecalis*, *Candida parapsilosis* and *Aspergillus fumigatus* were assessed using a surface time-kill test carried out on glass surfaces, with or without bovine serum albumin (BSA).

**Results:**

The bactericidal activity of the overheated dry-saturated steam vapour system was observed at 180 °C after 5 min contact with or without BSA, using an initial inoculum of 10^9^ CFU/mL. To reduce *C. parapsilosis* and *A. fumigatus* counts (from 10^7^ CFU/mL), a longer contact time was necessary (7 min). In vitro tests with sodium hypochlorite at 5 % in the absence of an organic substance also resulted in an overall reduction in bacterial counts (from 10^9^ CFU/mL) after 5 min of treatment. For mycotic challenge (10^7^ CFU/mL), a longer contact time was necessary (7 min). In the presence of an organic substance, after 5 min, the hypochlorite reduced the viable count from 10^9^ to 10^5^ CFU/mL for all bacterial strains except *E. faecalis* that showed a reduction of 2 log units (10^9^ to 10^7^ CFU/mL). For *C. parapsilosis* and *A. fumigatus*, a 2 log unit reduction was observed after 7 min.

**Conclusions:**

Steam disinfection of environmental surfaces using a portable steam generator is a practical and effective method that is not affected by the presence of organic matter.

## Background

Recent studies have demonstrated that several major nosocomial pathogens are shed by patients and contaminate environmental surfaces at concentrations sufficient for transmission [1–3]. Such pathogens can survive for extended periods despite cleaning with chlorine-releasing disinfectants [4, 5] and can be transferred to the hands of healthcare workers [6]. The spread of nosocomial pathogens has been linked to poor hand-hygiene practices. However, healthcare workers are more likely to contaminate their hands from touching the patient environment than from patient contact [6].

Mounting evidence demonstrates that outbreak strains of methicillin-resistant *Staphylococcus aureus* (MRSA), extensively drug-resistant (XDR) *Acinetobacter baumannii*, *Pseudomonas aeruginosa* and (extended spectrum beta-lactamase)-producing *Enterobacteriaceae* survived significantly longer on environmental surfaces than non-outbreak strains, indicating a possible fitness advantage [7–13].

In healthcare settings, surfaces are usually decontaminated using liquid chemical disinfectants, often chlorine derivatives [14]. However, these products have some drawbacks, they are usually toxic to humans, they display chemical reactivity, and they require long periods of contact (up to 15 min) with surfaces to kill microorganisms [15]. Moreover, some nosocomial pathogens are resistant to many disinfectants [16–18].

The development of portable steam generators has made the disinfection of environments more practical [19–21]. The aim of the present study was to assess “in vitro” the ability of the overheated dry-saturated steam vapour system to kill multidrug and XDR nosocomial pathogens on surfaces, and to define the antimicrobial spectrum and the contact times required by this system. Our results were then compared with those obtained using sodium hypochlorite, an agent commonly used in clinical sanitation procedures [14, 15].

## Methods

### Surfaces

The in vitro tests were carried out in a microbiology laboratory. Glass surfaces were chosen because they are flat, inert, easy to contaminate and highly resistant to chemical products and to heat.

The dimensions of the surfaces were 50 × 50 cm, with a thickness of 30 mm and a weight of 38 kg/m^2^.

### Disinfection using the overheated dry-saturated steam vapour system

**Fig. 1:**
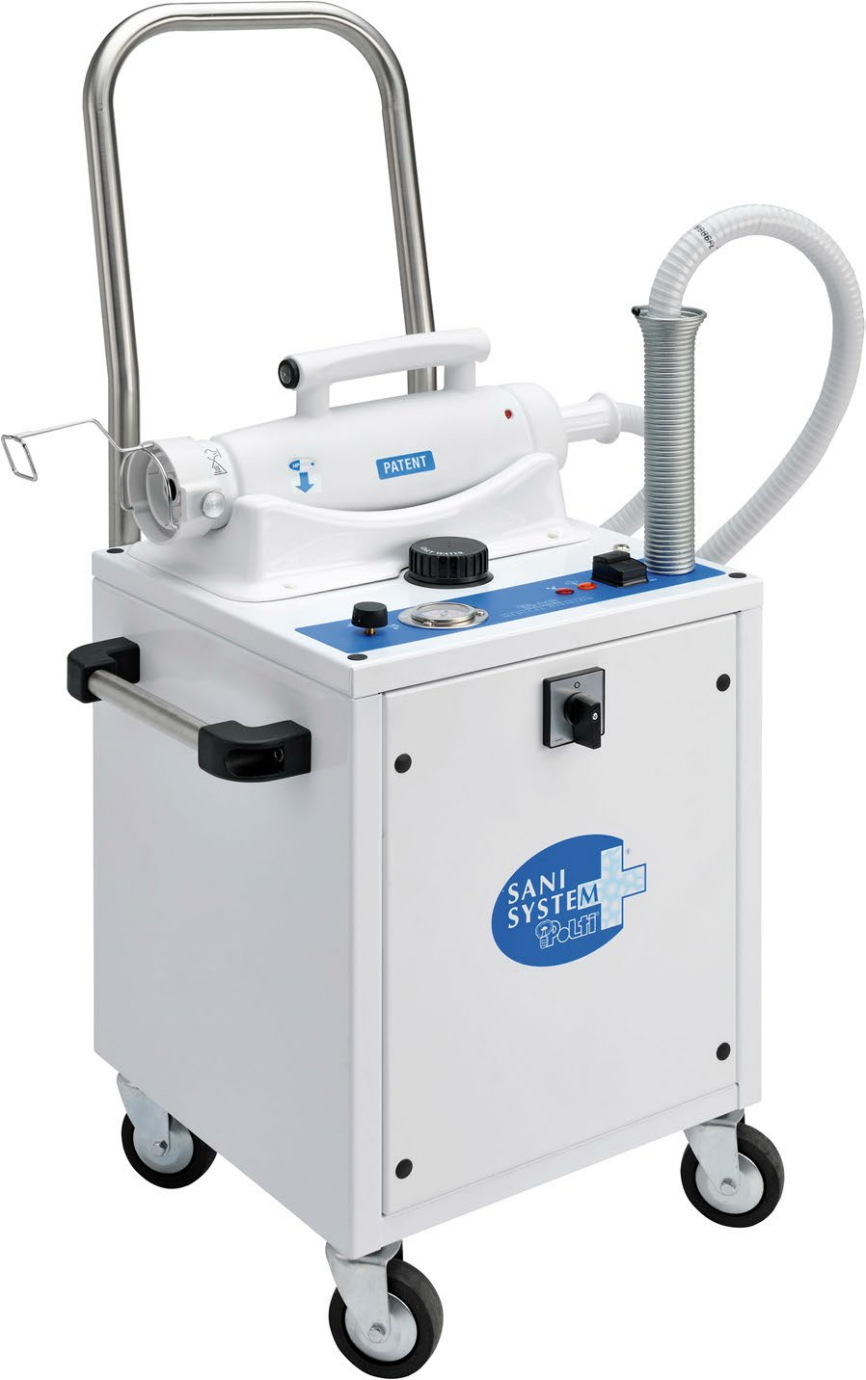
Overheated dry-saturated steam vapour disinfection device used in this study. (Sani System Polti, Medical Division Polti s.p.a., Como, Italy)

The steam generator device consists of a professional steam generator (Sani System Polti, Medical Division Polti s.p.a., Como, Italy) (Fig. 1). The dimensions of the unit were 47 × 45 × 90.5 cm, with a weight of 27.5 kg. The portable unit was outfitted with a hose connected to a steam dispenser. The overheated dry-saturated steam vapour was high temperature steam generated in the steel boiler that reached a pressure of 6 bar and was then further superheated in an expansion chamber to generate a dry saturated steam vapour at 180 °C. The unit was filled with tap water. Fifteen minutes before use, the unit was activated to reach the maximum operating boiler pressure (6 bar) in accordance with the manufacturer’s instructions (Sani System Polti). Protection equipment, such as heat-resistant gloves, safety aprons or glasses, is not required by workers when operating this system.

### Disinfection with sodium hypochlorite

The commercial product Decs containing sodium hypochlorite at 2.8 % (2.7 % active *chlorine)* (Lombarda H s.r.l, Albairate, Milan, Italy) was used in this study. The stock solution of the product was diluted in sterilised water to a final concentration of 5 % to obtain approximately 1400 ppm active chlorine. This is the concentration of sodium hypochlorite solution usually used for disinfection in hospitals [22].

### Culture methods

The bactericidal effects of the overheated dry-saturated steam vapour and of sodium hypochlorite were evaluated using seven environmental organisms, including Gram-positive and Gram-negative bacteria, yeast and fungi: XDR *A. baumannii* (strain 4500/2010), *P. aeruginosa* (strain 3637/2006), MRSA (strain 3582/2006), high-level aminoglycoside-resistant (HLAR) *Enterococcus faecalis* (strain 3084/2005), carbapenemase-producing *Klebsiella pneumoniae* (KPC; strain 4640/2012), *Candida parapsilosis* (strain 4093/2009) and *Aspergillus fumigatus* (strain 3430/2006). These environmental strains were isolated between January 2005 and December 2012 in the neonatal and the adult intensive care units of the University Hospital “Federico II” in Naples, Italy, during environmental microbiological investigations performed to identify sources and reservoirs of infection in the course of nosocomial outbreaks [10, 11].

Environmental isolates were identified by commercial systems (VITEK^®^ 2 automatic system; bioMèrieux Marcy-L’Etoile, France and Becton–Dickinson Phoenix, Phoenix Technologies Ltd, San Jose, CA, USA). All isolates were stored at −80 °C in glycerol solution.

### Susceptibility testing and screening

The strains used in our study were selected because of their antimicrobial resistance phenotypes.

Antimicrobial susceptibility patterns were analysed using an automated system (BD Phoenix) and by manual methods (i.e., Kirby-Bauer disk diffusion assay, Etest and microdilution tests) and the results were interpreted according to EUCAST [23]. Antimicrobial susceptibility testing revealed a multidrug-resistant antibiotype for all of the isolated microorganisms. The antimicrobial susceptibility patterns of strains included in the study are listed in Tables 1 and 2.

**Table 1:** Antimicrobial susceptibility patterns of the strains tested in this study

Antibiotic	MIC value					
	*KPC*-*K. pneumoniae*	*XDR A. baumannii*	*P. aeruginosa*	*E. faecalis HLAR*	*C. parapsilosis*	*A. fumigatus*
Amikacin	<4	>32	>32			
Amoxicillin-clavulanate	>16/8	>16	>32			
Ampicillin	>16		>32			
Ampicillin-sulbactam	>16/8					
Aztreonam	>16					
Cefazolin	>16		>64			
Cefepime			2			
Cefotaxime	>16	>32	>64			
Ceftazidime	>32	>16	>16			
Ceftriaxone	>32					
Chloramphenicol	16	>16				
Ciprofloxacin	>2	>2				
Gentamicin	≤2	>8	>8			
Gentamicin high-level				>2000		
Imipenem	8	>8	>8			
Levofloxacin	≤1	>2	>2			
Meropenem	8	>8	>8			
Netilmicyn high-level				>2000		
Nitrofurantoina	≤16		>512			
Norfloxacina	≤2					
Piperacillin	>64	>64	16			
Piperacillin–tazobactam	≥64/4	≥64/4	16			
Streptomycin high-level				>2000		
Tetracycline	>8	>8				
Trimethoprim–sulfamethoxazole	≤0.5/9.5	>2/38				
5-Flucytosine					>32^a^	
Fluconazole					>64^a^	
Itraconazole					>1^a^	>1^a^
Anidulafungin					>2^a^	>2^a^

^a^Sensititre Yeastone

**Table 2:** Disk diffusion susceptibility test and Etest values of *K. pneumoniae* strain identified as a KPC producer

Double disk test (mm zone diameter)				MIC of drug Etest value (mg/L)	
MEM (10 µg)	MEM plus boronic acid (600 µg)	MEM/MEM boronic acid	Interpretation	Ertapenem	Interpretation
16	21	>4	+	>1	+

#### In vitro time-kill tests

The bactericidal effects of the overheated dry-saturated steam vapour and the sodium hypochlorite on multidrug-resistant nosocomial pathogens were assessed by measuring viable cell counts using the quantitative time-kill test [24] as recommended by the European Committee for Standardization (CEN EN1276) with or without bovine serum albumin (BSA 0.3 g/100 mL) [25]. The quantitative time-kill test was performed as previously described [24]. In brief, a logarithmic-phase culture was adjusted to ca. 10^9^ CFU/mL for bacteria and 10^7^ CFU/mL for yeasts and fungi in Luria–Bertani broth. The test was carried out on glass surfaces initially contaminated with 100 μL of microbial suspension, prepared as above, with and without BSA. The surfaces were sanitised with the overheated dry-saturated steam vapour generated at 180 °C at different time points (1, 2, 3 min, up to 8 min). At each time point, the surfaces were rinsed with 1 mL of a sterile physiological solution, and 100 μL was removed from the rinsing solution and serially diluted (1:10, 1:100, 1:1000, 1:10,000) in phosphate-buffered saline (pH 7.4) and spread on Bacto D/E neutralizing agar (Becton–Dickinson). Plates were incubated at 37 °C for 24 h for bacterial strains and at 32 °C for 72 h for *C. parapsilosis* and *A. fumigatus* strains. Each experiment was performed in triplicate.

Time-kill tests following hypochlorite disinfection were carried out on glass surfaces with and without BSA. The glass surfaces were contaminated at eight different points with 100 μL of each microbial suspension. The surfaces were sanitised at each inoculum point with 900 µL of disinfectant solution at room temperature (22 °C) and then 100 µL of each suspension was removed from the surfaces at different time points and spread on Bacto D/E neutralizing agar after serial dilution, as described above. Plates were incubated under the same conditions as above. The antimicrobial action of chlorine was neutralised by sodium thiosulphate [26], which is contained within the D/E neutralizing agar at a concentration of 6.0 g/L. Each experiment was performed in triplicate.

Viable cell counts of the bacterial, yeast and fungal strains were evaluated on agar plates (Bacto D/E neutralizing agar) after incubation under the conditions described above. The results of the in vitro tests were interpreted in accordance with the CEN standards [25]. According to these standards, the bactericidal activity of disinfectant was defined as a ≥4 log 10 CFU/mL decrease in the viable count compared with the initial inoculum.

### Statistical analysis

Data were analysed using GraphPad Prism v.5.04 software (GraphPad Software, La Jolla, CA, USA). The significance of the reduction in viable counts was analysed using the Student’s *t* test and the reduction was considered significant when the P value was <0.05.

## Results and discussion

### Overheated dry-saturated steam vapour

**Fig. 2:**
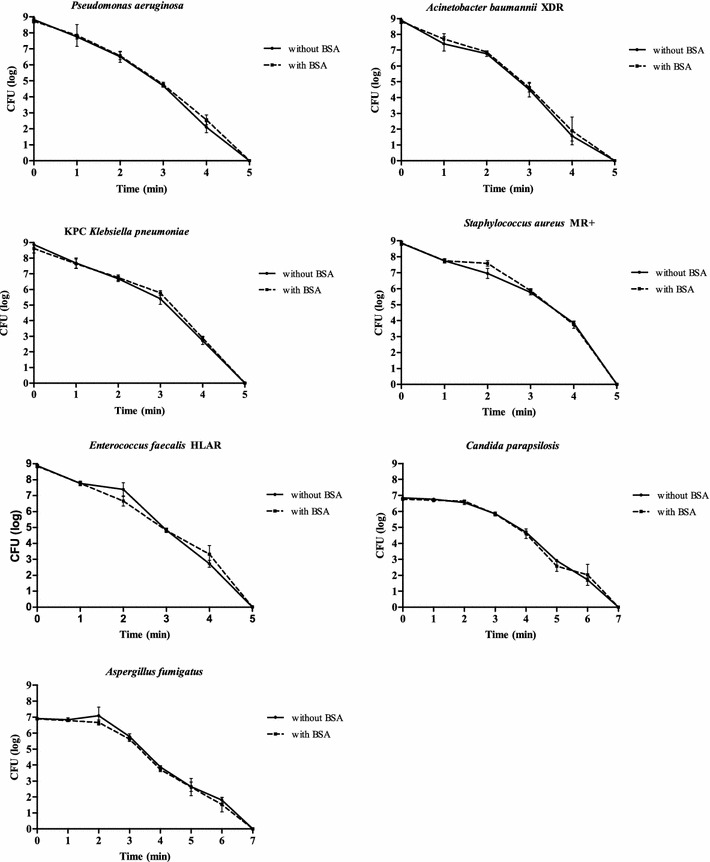
Microbial time-kill diagrams after dry saturated steam vapour treatment with or without BSA

The bactericidal activity of the dry saturated, steam vapour system was observed for Gram-negative bacteria at a temperature of 180 °C after 5 min of treatment without BSA. A significant reduction in *P. aeruginosa* (p = 0.0004, as determined by the Student’s t test compared with untreated samples), XDR *A. baumannii * (p < 0.0001) and KPC (p < 0.0001), from an initial concentration of 10^9^ CFU/mL, was observed after 2 min of treatment with this system. The same results were obtained in the presence of BSA (Fig. 2). For MRSA and HLAR *E. faecalis*, bactericidal activity was observed after 5 min contact time with a significant reduction (p < 0.0001) from the initial concentration (10^9^ CFU/mL) after 3 min of treatment, in the presence and absence of BSA (Fig. 2).

A longer contact time (7 min) was necessary to reduce the viable count of *C. parapsilosis* and *A. fumigatus* (to 10^7^ CFU/mL) without or with BSA, although a significant reduction in the yeast (p < 0.0001) and fungi (p = 0.0004) counts from the initial concentration (10^7^ CFU/mL) was observed after 3 min of treatment (Fig. 2).

### Sodium hypochlorite

**Fig. 3:**
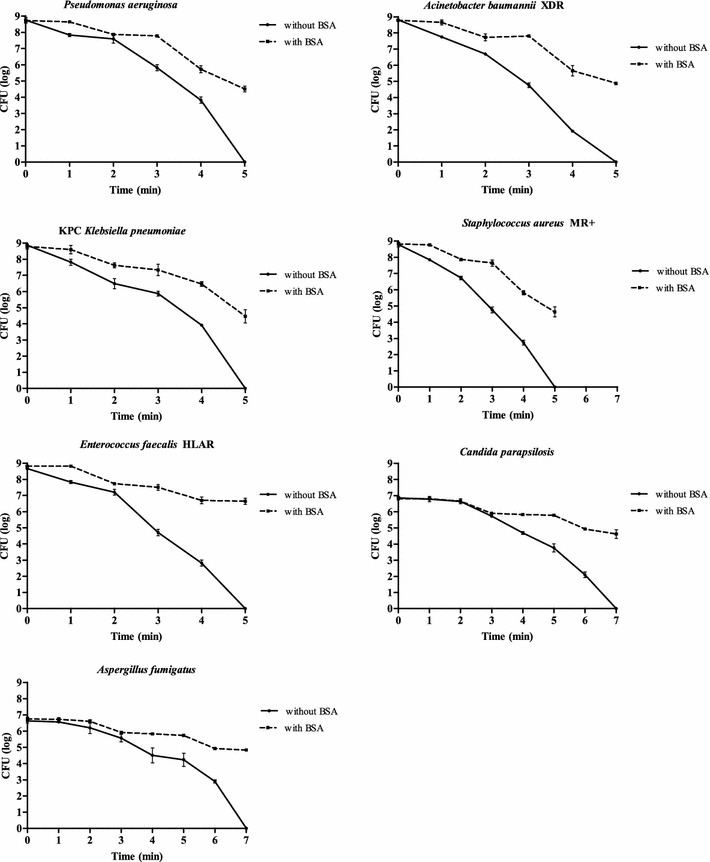
Microbial time-kill diagrams after chlorine-derivative treatment with or without BSA

The in vitro tests with sodium hypochlorite at 5 % in the absence of an organic substance (i.e. BSA) resulted in an overall reduction in the viable count within 5 min for Gram-negative and Gram-positive bacteria (from 10^9^ CFU/mL) (Fig. 3). A longer contact time (7 min) was necessary under the same conditions without BSA to reduce the yeast and fungi count (from 10^7^ CFU/mL) (Fig. 3).

Conversely, 5-min hypochlorite treatment in the presence of an organic substance reduced the initial viable count (10^9^ CFU/mL) to 10^5^ CFU/mL (4 log decrease) for all bacterial strains, except HLAR *E. faecalis* that showed a 2 log decrease (10^9^ to 10^7^ CFU/mL). The reduction in *C. parapsilosis* and *A. fumigatus* counts (10^7^ to 10^5^ CFU/mL) at 7 min was only 2 log units in the presence of BSA (Fig. 3).

The disinfection of all multidrug-resistant pathogens was rapid and complete. This is an important result because surface-related transmission of antibiotic-resistant microorganisms is a growing threat in healthcare settings and chemical disinfectants may have the potential to select cross-resistance to antibiotics [16, 27].

The in vitro tests using sodium hypochlorite at 5 % in the absence of an organic substance also resulted in an overall reduction in bacterial and mycotic concentrations. In the presence of an organic substance, the antimicrobial activity of sodium hypochlorite was reduced. The use of disinfectants requires an initial cleaning step to remove the organic matter that would otherwise “consume” the oxidizing disinfectant [14, 15]. Therefore, in the presence of an organic substance, a large quantity of oxidising disinfectant (containing chlorine and ozone) may be required, often at higher-than-standard concentrations. The disinfecting effect of chlorine is only visible when the “requirement” of organic substances has been met [14, 15]. In contrast, the steam vapour device depends on heat for efficacy and the presence of organic matter does not influence the effectiveness [28]. Moist heat acts by denaturation and coagulation of protein, breakage of DNA strands and loss of functional integrity of cell membranes, resulting in cell death [29].

This system could help to reduce the risk of spreading nosocomial infections in healthcare facilities.

Our time-kill curve studies revealed the time-dependent effect of killing by steam vapour disinfection with an overall drop in microbial counts obtained at either 5 or 7 min depending on the microorganism. These findings are in accordance with previous studies performed using saturated steam showing that this method is effective in decontaminating surfaces contaminated with high concentrations of pathogenic microorganisms, killing 100 % of bacteria under all experimental conditions [19–21]. The antimicrobial activity of the steam disinfection system has great potential in the disinfection of contaminated hospital environments, because many of the most important nosocomial pathogens, such as *P. aeruginosa* and *A. baumannii*, have natural resistance to liquid chemical disinfectants and especially to quaternary ammonium compounds [30]. The saturated steam vapour disinfection system has a broad range of activity [19–21] and this technology is also considered to have great potential for eliminating biofilms, aggregates of active cells embedded within a polymeric matrix and attached to a biotic or abiotic surface [31].

## Conclusions

The steam vapour system has been proven to reduce or completely eliminate microbial contamination on hard surfaces at earlier and later time points, respectively. This portable device quickly reduced and then eliminated microbial loads in the presence or absence or an organic substance in contrast to sodium hypochlorite, whose disinfectant effect is only visible when the “requirement” of the organic substance has been met. The findings of this study suggest that the portable vapour disinfection system is a viable alternative to available chemical disinfectants, including chloride derivatives, for the disinfection of hospital environmental surfaces.
